# Influence of Age on Anticontractile Effect of Perivascular Adipose Tissue in Normotensive and Hypertensive Rats

**DOI:** 10.1155/2019/9314260

**Published:** 2019-01-17

**Authors:** Anna Zemančíková, Jozef Török

**Affiliations:** Centre of Experimental Medicine, Institute of Normal and Pathological Physiology, Slovak Academy of Sciences, Bratislava, Slovakia

## Abstract

Perivascular adipose tissue (PVAT) and its vasomodulatory effects play an important role in the physiology and pathophysiology of blood vessels. Alterations in PVAT associated with reduction in its anticontractile influence are proven to contribute to vascular dysfunction in hypertension. The aim of this study was to examine whether the changes in PVAT properties could participate in progression of vascular abnormalities in developing spontaneously hypertensive rats (SHR). Normotensive Wistar-Kyoto (WKY) rats and SHR, both in 5th and in 12th week of age, were used. Systolic blood pressure was similar between WKY rats and SHR in 5th week of age; however, in 12th week, it was significantly increased in SHR comparing to WKY rats. The amount of retroperitoneal fat was higher in WKY rats in both age groups, whereas body weight was higher in WKY rats only in 12th week, when compared to age-matched SHR. From isolated superior mesenteric arteries, two ring preparations were prepared for isometric tension recording, one with PVAT intact and other with PVAT removed. In WKY rats as well as in SHR, arterial contractile responses to noradrenaline, applied cumulatively on rings, were significantly inhibited in the presence of intact PVAT. In both age groups, anticontractile effect of PVAT was higher in WKY rats than in SHR. Neurogenic contractions, induced by electrical stimulation of perivascular sympathoadrenergic nerves, were significantly attenuated in the presence of PVAT in WKY mesenteric arteries from both age groups; however, in arteries from SHR, intact PVAT had no influence on this type of contractile responses. The results suggest that in SHR impairment of anticontractile effect of PVAT precedes hypertension and might contribute to its development.

## 1. Introduction

Perivascular adipose tissue (PVAT) has recently attracted high attention in cardiovascular research. Since the period of first experimental findings regarding the anticontractile effect of PVAT [1] and the potential existence of PVAT-derived relaxing factor(s) [2], many data have been gathered about biologically active substances of various chemical origin which are produced in PVAT and are capable of influencing vascular tone, physiological and pathophysiological growth of particular components of vessel wall, function of vascular autonomic nerves, cell migration, or inflammation processes [3–5]. One of the most studied effects of PVAT is its anticontractile influence which is probably mediated by several PVAT-derived factors; the most important seem to be angiotensin 1-7, methyl palmitate, hydrogen sulphide, nitric oxide, hydrogen peroxide, etc. These substances act as hyperpolarizing factors which increase the membrane potential of vascular smooth muscle cells by activating different types of potassium channels, and this process leads to inhibition of vasoconstriction [4]. The mentioned findings refer to close relationship between PVAT and the proper vascular wall, which determines the correct development and function of vessels, and its disruption might be the cause as well as the consequence of various vascular pathologies. In clinical practice, the occurrence of vasospasms is well documented when PVAT is removed during isolation of vessel grafts before their chirurgical use [6]. On the other hand, in pathological conditions associated with cardiovascular and metabolic dysfunction, adipocytes might be an important source of free radicals and proinflammatory cytokines [7]. These molecules lead to further enhancement of oxidative stress, through uncoupling of endothelial nitric oxide synthase and production of peroxynitrite radical instead of nitric oxide. In addition, macrophages and T lymphocytes infiltrate adipose tissue due to oxidative stress activation [8]. It was shown that in obese patients with metabolic syndrome, the total PVAT mass around small arteries was increased while its anticontractile effect was completely lost, and markers of hypoxia and inflammation were detected in this compartment [9]. Similar results were found also in animal genetic and diet-induced models of obesity [10, 11]. On the other hand, the impairment in anticontractile action of mesenteric arterial PVAT was detected also in spontaneously hypertensive rats (SHR), which are characteristic by lower body weight comparing to normotensive Wistar-Kyoto rats [12], and this defect in PVAT was shown to be associated with the exaggerated sensitivity of SHR arteries to vasoconstrictors [13, 14]. Moderate growth in adipose mass due to high fructose intake potentiated the anticontractile properties of PVAT in normotensive rats but it did not improve them in SHR, indicating that qualitative rather than quantitative changes of PVAT are important when considering its participation in increased vascular tone in SHR [15].

The abovementioned data show that the role of PVAT in physiology and pathophysiology of vascular system is quite well documented in adult individuals. However, little is known about the function of PVAT during growth and development of cardiovascular system in healthy subjects as well as in those with predisposition to cardiovascular dysfunction. Therefore, the aim of this study was to compare the anticontractile effect of PVAT on adrenergic responses of mesenteric arteries in juvenile and adult rats of normotensive and hypertensive strain.

## 2. Materials and Methods

### 2.1. Experimental Animals

Male normotensive Wistar-Kyoto (WKY) rats and spontaneously hypertensive rats (SHR), both strains at 5th and at 12th week of age, were used. All rats were born in our certified animal facility (Institute of Normal and Pathological Physiology, Centre of Experimental Medicine SAS), housed at temperature 22–24°C and humidity (45–60%) with a 12 : 12 h light-dark cycle (lights on from 06.00 a.m. to 06.00 p.m.), and fed with a standard pellet diet with tap water ad libitum. One day before experiment, their systolic blood pressure was measured in conscious state by the noninvasive tail-cuff method. Food was withdrawn 12 hours before the experiment. Rats were sacrificed under CO_2_ anesthesia; their heart, liver, and retroperitoneal fat were weighed, and superior mesenteric artery was removed and prepared for isometric tension recording.

The animal protocols used in this study were performed in accordance with the *Guide for the Care and Use of Laboratory Animals* published by the National Institutes of Health and approved by the Animal Health and Welfare Division of the State Veterinary and Food Administration of Slovakia.

### 2.2. Functional Studies on Isolated Mesenteric Arteries

Superior mesenteric artery was isolated from rat, quickly transferred to cold Krebs solution, and dissected into paired endothelium-intact rings (2.8–3.2 mm in length) — one with PVAT preserved and other with PVAT removed. In the case of PVAT−rings (without PVAT), under a microscope, the perivascular fat was removed from arterial surface with fine scissors, with caution not to damage the adventitia. In the case of PVAT+ rings (with PVAT), a continuous layer of perivascular fat (1 to 1.2 mm in width) was left around the vessel. Each arterial preparation was set up for isometric tension recording using a force-displacement transducer Sanborn FT 10 (Sanborn, Baltimore, USA) and suspended in 20 ml organ baths filled with oxygenated (95% O_2_ + 5% CO_2_) modified Krebs solution maintained at 37°C. The Krebs solution was prepared in the following composition (in mmol/l): NaCl 118, KCl 5, CaCl_2_ 2.5, MgSO_4_ 1.2, NaHCO_3_ 25, KH_2_PO_4_ 1.2, glucose 11, and CaNa_2_.EDTA 0.03. The preparations were equilibrated under a resting tension of 10 mN for 60–90 min, and the Krebs solution was changed every 15 min.

Adrenergic contractions were registered in mesenteric arteries as the responses to cumulatively applied exogenous noradrenaline (increasing concentrations were applied in a cumulative manner) or as the neurogenic responses elicited by electrical stimulation of periarterial sympathetic nerves. The arterial rings were stimulated by two parallel platinum plate electrodes placed on either side of the preparation and connected to an electrostimulator ST-3 (Hungary). Frequency-response curves to electrical stimuli were obtained using square pulses of 0.5 ms in duration, at supramaximal voltage (>30 V), applied at 1–32 Hz, for a period of 20 s. In our previous unpublished observations, we found that the contractions of rat mesenteric arteries elicited by transmural electrical stimulation (TES) using the described parameters of stimulation, are blocked by phentolamine or tetrodotoxin, indicating that they are induced mainly by nerve-released noradrenaline [16].

In mesenteric arteries, contractions to 100 mmol/l KCl were also determined.

### 2.3. Data Analysis

Arterial isometric contractile responses were expressed as the active wall tension in mN and normalized to the length of respective ring preparation (mm). Area under curve (AUC, in arbitrary units) was calculated from individual concentration/frequency-response curves in each experimental group. The negative log10 concentration required to achieve the half-maximum contraction (pEC50) was calculated for each dose-response curve to express the sensitivity to noradrenaline.

Statistical evaluation was carried out by using one-way analysis of variance (ANOVA). The results were considered to be significant when *p* < 0.05.

## 3. Results

In 5th week of age, SHR differed from WKY rats in higher heart rate and relative heart weight, as well as in lower amount of retroperitoneal fat. These differences were detected also in 12th week of age; moreover, 12-week-old SHR have significantly higher blood pressure and lower whole body weight, in comparison with age-matched WKY rats (Table 1).

**Figure 1:**
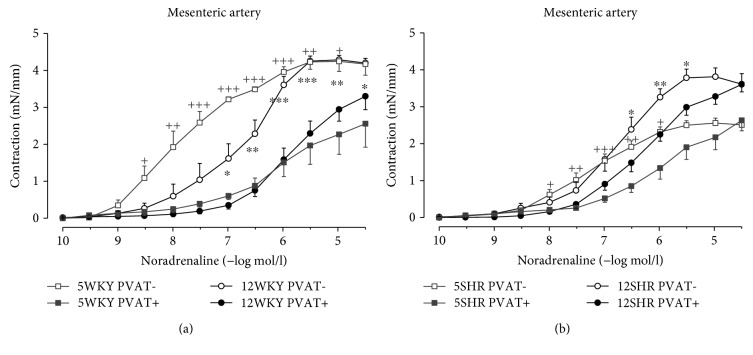
Dose-response curves to exogenous noradrenaline in mesenteric arteries with intact (+) and removed (−) perivascular adipose tissue (PVAT) from (a) 5- and 12-week-old Wistar-Kyoto rats (5WKY and 12WKY) and (b) 5- and 12-week-old spontaneously hypertensive rats (5SHR and 12SHR). Values represent mean ± SEM of 10 rats. ^+^*p* < 0.05, ^++^*p* < 0.01, and ^+++^*p* < 0.001 PVAT− vs. PVAT+ arteries from 5WKY and 5SHR; ^∗^*p* < 0.05, ^∗∗^*p* < 0.01, and ^∗∗∗^*p* < 0.001 PVAT− vs. PVAT+ arteries from 12WKY and 12SHR.

Contractile responses of mesenteric arteries to exogenous noradrenaline were attenuated in the presence of PVAT in WKY rats as well as in SHR. However, the anticontractile effect of PVAT was higher in WKY rats than in SHR in both ages, as seen from the differences in AUCs for noradrenaline dose-response curves between PVAT− and PVAT+ arterial rings (AUC_PVAT−_ − AUC_PVAT+_) in 5th week of age (19.23 ± 2.67 in WKY rats vs. 4.91 ± 1.23 in SHR; *p* < 0.01) and in 12th week of age (10.86 ± 2.58 in WKY rats vs. 4.49 ± 1.01 in SHR; *p* < 0.05). The contractile characteristics demonstrated in Table 2 and the whole dose-response curves of contractile responses to noradrenaline (Figure 1(a)) indicate that mesenteric arterial preparations with intact PVAT respond similarly in 5- and in 12-week-old WKY rats; however, in PVAT-denuded rings, 5-week-old WKY rats exhibit significantly higher sensitivity to noradrenaline (pEC50; *p* < 0.01) with no difference in maximum contraction, when compared to 12-week-old WKY rats (Figure 1(a)). On the other hand, in 5-week-old SHR, the dose-dependent noradrenaline contractions in PVAT− as well as in PVAT+ mesenteric arteries showed smaller AUC values and maximal tension when compared to 12-week-old individuals (Table 2; Figure 1(b)).

**Figure 2:**
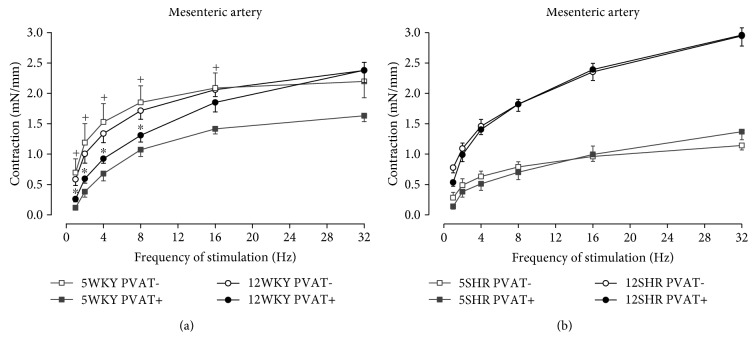
Frequency-response curves to transmural electrical stimulation in mesenteric arteries with intact (+) and removed (−) perivascular adipose tissue (PVAT) from (a) 5- and 12-week-old Wistar-Kyoto rats (5WKY and 12WKY) and (b) 5- and 12-week-old spontaneously hypertensive rats (5SHR and 12SHR). Values represent mean ± SEM of 10 rats. ^+^*p* < 0.05 PVAT− vs. PVAT+ arteries from 5WKY; ^∗^*p* < 0.05 PVAT− vs. PVAT+ arteries from 12WKY.

Neurogenic contractions of mesenteric arteries, induced by endogenous noradrenaline released from perivascular sympathetic nerves by electrical stimulation (frequency-dependent), were decreased due to the presence of intact PVAT only in mesenteric arteries from WKY rats, similarly in both age groups, as shown in Figure 2(a). The anticontractile effect of PVAT was manifested by the reduction in AUC only in 5-week-old WKY rats (Table 2). The inhibitory effect of PVAT on neurogenic contractions was not shown in SHR. The TES-induced contractions were significantly smaller in 5-week-old SHR comparing to 12-week-old SHR, in PVAT− as well as in PVAT+ mesenteric arterial preparations (Figure 2(b)).

**Figure 3:**
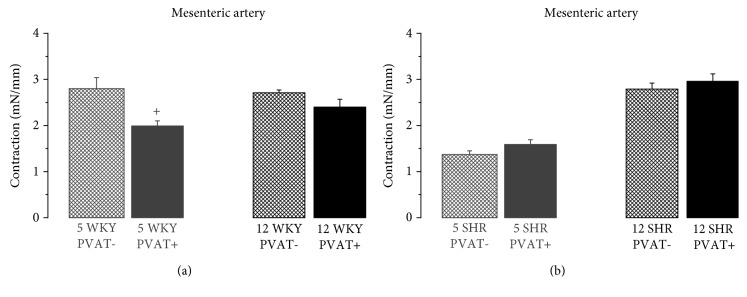
Contractile responses to excitation by high K^+^ concentration in bath solution (100 mmol/l KCl) in mesenteric arteries with intact (+) and removed (−) perivascular adipose tissue (PVAT) from (a) 5- and 12-week-old Wistar-Kyoto rats (5WKY and 12WKY) and (b) 5- and 12-week-old spontaneously hypertensive rats (5SHR and 12SHR). Values represent mean ± SEM of 10 rats. ^+^*p* < 0.05 PVAT− vs. PVAT+ arteries from 5WKY.

Contractile responses of mesenteric arteries to excitation by high K^+^ concentration in bath solution (100 mmol/l KCl) were reduced in the presence of PVAT only in 5-week-old WKY rats (Figure 3).

## 4. Discussion

Perivascular adipose tissue has recently been considered by several authors as an integral part of vessels (*Tunica adiposa*) as it can directly participate on their functions—it regulates the vasomotorics, growth, and immunological processes in the vascular wall [17]. In some vessels, this tissue is not separated from the proper wall and directly follows the adventitial layer; in others, it is distinctly detached and easily separable. This indicates that PVAT might have various functions in different vessels, which is confirmed also by the variability in its composition (white, brown, and beige: white PVAT with admixture of brown adipocytes), in the presence of cells of the immune system, and in the character and density of its innervation [4, 18, 19]. From our presented results, it seems that PVAT might play also an important role during the ontogenesis of vascular system, with respect to the normal setup of vascular functions as well as to the origin of various vessel pathologies during the development of cardiovascular disorders.

In the present study, PVAT exhibited an inhibitory effect on contractile responses to exogenous noradrenaline in superior mesenteric arteries from both normotensive and (pre)hypertensive rats in 5th as well as in 12th week of life. However, within both age groups, this effect was significantly weaker in SHR than in WKY rats. From the ontogenetic point of view, the anticontractile action of PVAT was stronger in 5th week than in 12th week in WKY arteries, whereas in SHR, there was no significant difference in magnitude of PVAT effect between 5th and 12th week of life.

In WKY rats, making comparison between 5th and 12th week of their life, there was a marked shift in sensitivity of mesenteric arterial smooth muscle to noradrenaline, which was visible only after elimination of the inhibitory influence of PVAT; in the presence of PVAT, there was no significant difference in this reaction between young and adult WKY rats. After removing PVAT from the surface, mesenteric arteries from 5-week-old WKY rats showed atypical (non-S-shaped) dose-response curve with high sensitivity to lower doses of noradrenaline; however, the maximal contraction in response to highest noradrenaline concentrations was similar between 5- and 12-week-old WKY rats (Figure 1(a)). From these observations, it is possible to presume that the proper contractile apparatus of mesenteric arterial smooth muscle (its contractile capacity) is already well developed in 5-week-old WKY rats; on the other hand, its adrenergic regulation including the feedback mechanisms (structure, quantity, and spatial distribution of synapses, adrenoreceptors, and other associated regulatory proteins in smooth muscle cells as well as in sympathoadrenergic nerve terminals) at this age is probably still developing, and the particular regulatory components and mechanisms are not adequately set up yet. The noradrenaline dose-response curve with high EC50 value in arteries from young WKY rats indicates that low level of sympathetic stimulation could evoke the excessive contractile response which might lead to the strong increase in vascular resistance. The comparison of noradrenaline dose-response reactions between PVAT-intact and PVAT-removed arterial preparations showed that PVAT with its inhibitory influence substantially attenuates the exaggerated contractile response to low noradrenaline concentrations in arteries from young WKY rats, in which the adrenergic regulatory mechanisms and their proper setting up are still under development. From the abovementioned discussion, it follows that the anticontractile effect of PVAT plays an important role during the development of vascular system and its regulations in normotensive rats; this was evident in the contractile responses to exogenous (Figure 1(a)) as well as to endogenous noradrenaline (Figure 2(a)) released from electrically excited sympathetic nerves in the proper vascular wall.

The considerable anticontractile influence of PVAT in mesenteric arteries from 5-week-old WKY rats was also manifested by attenuation of contractile responses to depolarization by high extracellular K^+^ (Figure 3). In this case, the K^+^ channel-mediated hyperpolarizing effect of PVAT-derived relaxing factor(s) should be prevented; however, other mechanisms mediating the anticontractile effect could be involved, e.g., calcium desensitization of the arterial contractile apparatus through decreasing Rho kinase activity. This mechanism might even potentiate the anticontractile effect of PVAT in mesenteric arteries from young WKY rats when the sensitivity of vascular smooth muscle to vasoconstrictors is excessively high.

Similar effect in reaction to exogenous, however not to endogenous noradrenaline, was shown in PVAT-free mesenteric arteries from 5-week-old SHR, which exhibited flatter dose-response curve (Figure 1(b)) with higher EC50 (Table 2) in comparison with preparations from 12-week-old SHR. The analysis of arterial dose-dependent responses to noradrenaline revealed the differences in arterial contractile responses between 5-week-old SHR and WKY rats which were detectable only after eliminating the inhibitory effect of PVAT: in these conditions, the arterial sensitivity to noradrenaline was increased in both strains; however, SHR arteries produced lower contractile force in response to particular doses of noradrenaline (Figure 1(b)) as well as to frequency-dependent sympathoneural excitation during TES (Figure 2(b)). Such finding could be caused by thinner arterial wall in young, prehypertensive SHR compared to age-matched WKY rats, which is documented by morphological findings in the study made by Török et al. [20]. It seems that in 5-week-old SHR, the growth and development of arterial contractile components lag behind, but they catch up relatively fast during the next weeks of development, probably due to increasing trophic influence of pressure overload as well as due to the excess noradrenaline levels occurring with sympathetic hyperinnervation. Contrarily, in the presence of PVAT, the characteristics of noradrenaline dose-response curves did not significantly differ between mesenteric arteries from 5-week-old SHR and WKY rats; it can be presumed that in such conditions the contractile responses are equalized by stronger anticontractile effect of PVAT in arteries from WKY rats together with smaller contractile force in arteries from SHR.

In contrast to the 5th week of life in which the marked differences in noradrenaline responses between WKY and SHR mesenteric arteries were detectable only after removal of PVAT, at 12th week of life, the situation seemed to be inverse: in PVAT-free arterial preparations, the contractions were very similar between WKY rats and SHR, while the differences in sensitivity to noradrenaline manifested only in conditions of preserved PVAT. In PVAT-intact arteries, the sensitivity to noradrenaline is higher in SHR which indicates that the anticontractile effect of PVAT in SHR mesenteric arteries is significantly reduced comparing to age-matched WKY rats. Moreover, from the abovementioned discussion, it follows that PVAT importantly participates on inhibiting the arterial contractions in adult normotensive WKY rats, and that the “anticontractile dysfunction” of PVAT could be involved — among other factors — in enhanced arterial sympathetic responses in hypertensive rats.

The question remains about the density of arterial sympathetic innervation, its developmental state at 5th week of age in WKY rats and in SHR, and its distribution between the proper vascular wall and PVAT, as well as about endothelial regulation, its developmental aspects, and its dysfunction in hypertension. However, the interaction of so many factors is difficult to consider simultaneously, and simplification is needed. Nevertheless, it is appropriate to mention some of the available literary data as well as some of our previous observations which suggest that, e.g., the endothelium-dependent relaxant responses are well established and equal between 5-week-old WKY rats and SHR [20], whereas in older individuals, these responses were found to be impaired [12] or preserved [21] in hypertensive comparing to normotensive rats. The regulatory influence of endothelium and PVAT on vascular smooth muscle seems to be largely independent, although several authors brought some evidence that substances released from PVAT could provoke or block the release and impact of some vasoactive factors from endothelium and vice versa [22, 23].

Regarding the sympathetic innervation, in our recent study, we have found that in adult normotensive rats, most of the sympathetic nerve terminals are concentrated in the surface layers of the proper mesenteric arterial wall, and the active innervation is only sparsely present in PVAT. This is supported by the finding of significant increase in neurogenic contractions of WKY mesenteric arteries after removal of PVAT, which is caused by elimination of anticontractile influence of PVAT, whereas most of the sympathetic nerve terminals remained to be preserved in arterial wall [14]. Similar observations were obtained in the present study with 5-week-old WKY rats in which the anticontractile effect of PVAT was even stronger than in adult rats — the difference between reactions from arteries with PVAT intact and removed was greater (Figure 1(a)).

On the other hand, after removal PVAT from the surface of WKY mesenteric arteries, there was no significant difference in neurogenic contractions between 5th and 12th weeks of age (Figure 2(a)). This seems to be in contrast with the abovementioned high sensitivity to exogenous noradrenaline in PVAT-free mesenteric arteries from 5-week-old WKY rats comparing to 12-week-old WKY rats. Such findings indicate that in young WKY rats, the function of probably not yet enough developed sympathetic innervation is compensated by the hypersensitivity of the adrenergic system. It might be supposed that PVAT plays an important role as a buffer to equilibrate this imbalance — it significantly attenuates the possible excessive adrenergic contractions in arteries, particularly in young rats, which confirms its important role in growth and development of vascular system and in setup of its sympathetic regulation.

In mesenteric arteries of both 5- and 12-week-old SHR, the anticontractile effect of PVAT was not manifested during neurogenic contractions (Figure 2(b)). It is noteworthy that in these rats, PVAT was able to attenuate the contractile responses to exogenous noradrenaline but not to endogenous noradrenaline released from sympathetic nerve terminals from the proper arterial wall during TES. One of the explanations proposed is that in SHR mesenteric arteries, some procontractile substances are released during TES from PVAT, which overlap its anticontractile effect. Lu et al. [24] and Gao et al. [25] found that electrical stimulation provokes the release of angiotensin II and superoxide from PVAT which potentiate the neurogenic contractions in normotensive rats. In SHR, these substances are produced in increased amount [26, 27]; therefore, they could be even more abundantly released from PVAT during TES, and they might seemingly eliminate the anticontractile effect of PVAT. Moreover, an important role might play the increased density of sympathetic innervation in arterial wall, which is characteristic of SHR; in arteries from these rats, the multiplied sympathetic nerves may extend into PVAT which means that PVAT itself could be a source of endogenous noradrenaline released during TES [28]. All the mentioned factors can partially be causing that in SHR mesenteric arteries, the intact PVAT does not manifest anticontractile effect on neurogenic contractions, because during TES, besides inhibitory PVAT-derived factors, substances producing contraction might also be released from PVAT.

Spontaneously hypertensive rats usually have lower body weight and, correspondingly, lower content of body fat, when compared to control normotensive WKY rats; this is confirmed also by the results of this study. Gálvez et al. [13] found that the mesentery weight and its total lipid content are lower in SHR comparing to WKY rats. It is possible to presume that smaller amount of PVAT in SHR results in smaller quantity of anticontractile substances produced and released from PVAT. However, concerning the differences in PVAT between SHR and WKY rats, qualitative distinctions seem to be more important than the quantitative ones [29]. To support this concept, in our previous study, we have demonstrated that moderate increase in body adiposity potentiates the anticontractile effect of PVAT in normotensive WKY rats but not in SHR [15]. Therefore, it is evident that SHR have qualitatively different PVAT, and the increase in amount of this tissue might not cause its larger anticontractile effect.

Besides the mentioned qualitative distinctions in PVAT itself, moreover, the deficient function of K^+^ channels in vascular smooth muscle [30, 31] seems to contribute to the weaker anticontractile effect of PVAT in SHR. The K^+^ channels mediate the effect of most PVAT-derived relaxing factors by hyperpolarization of smooth muscle, and their impaired function in SHR could be reflected also in dysfunction of PVAT.

In summary, PVAT plays an important role in growth and development of vascular system and its sympathetic regulation in normotensive WKY rats. Perivascular fat in mesenteric arteries from SHR exhibits weaker anticontractile effect comparing to WKY rats, which could contribute to the increase in arterial resistance in SHR. Dysfunction of PVAT is already present in young individuals, but the smaller arterial smooth muscle thickness in 5-week-old SHR causes that reduced anticontractile effect of PVAT does not manifest by increased noradrenergic contractions, when compared to WKY arteries. The revealed distinctions in growth processes between vascular systems of young WKY rats and SHR, expressed, i.e., by smaller contractile capacity of vascular smooth muscle in young SHR, seem to be hidden and not detectable in the presence of intact PVAT which indicates its significance in physiology as well as in pathophysiology of the developmental processes in vascular system. It seems that in the period of life between 5th and 12th weeks, the substantial changes in rat mesenteric arterial reactivity mostly reflect the functional and morphological alterations in vascular smooth muscle and its innervation, whereas the PVAT anticontractile effect does not seem to undergo the major changes, although its importance is high in both age stages. In WKY rats at 5th week of age, the significantly increased attenuating effect of mesenteric arterial PVAT possibly dampens the exaggerated sensitivity of smooth muscle to adrenergic stimulation and enables its proper setting up, which might be mediated by different amount and spectrum of PVAT-released relaxing factors or by their increased efficacy in smooth muscle. Therefore, our results confirmed PVAT to be an important component of arterial wall and pointed out the importance of this structure in assessing the results of *in vitro* experiments with isolated vessels as well as during isolation and preparation of vascular grafts in clinical procedures.

## Figures and Tables

**Table 1:** General characteristics of experimental animals.

	WKY-5w	WKY-12w	SHR-5w	SHR-12w
BW (g)	98.3 ± 5.0	289.5 ± 2.3	91.0 ± 5.5	252.4±4.2^∗∗∗^
SBP (mm Hg)	110.5 ± 1.9	115.4 ± 0.9	115.6 ± 2.8	165.6±3.1^∗∗∗^
HR (bpm)	418.6 ± 15.9	351.5 ± 8.2	540.2±12.6^∗∗∗^	460.1±13.0^∗∗∗^
HW/TL (mg/mm)	15.5 ± 0.3	29.7 ± 0.3	16.8 ± 0.4^∗^	32.0 ± 1.0^∗^
LiW/TL (mg/mm)	145.7 ± 11.1	215.9 ± 3.3	169.1 ± 12.4	219.6 ± 7.3
RFW/TL (mg/mm)	10.9 ± 1.2	56.9 ± 3.3	7.0 ± 0.7^∗^	39.9±1.8^∗∗∗^

BW: body weight; SBP: systolic blood pressure; HR: heart rate; HW/TL: heart weight/tibia length; LiW/TL: liver weight/tibia length; RFW/TL: retroperitoneal fat weight/tibia length. Values represent mean ± SEM of 10 rats. ^∗^*p* < 0.05 and ^∗∗∗^*p* < 0.001 SHR-5w/12w vs. WKY-5w/12w (age-matched).

**Table 2:** Characterization of adrenergic contractions in mesenteric arteries with perivascular adipose tissue removed (PVAT−) and intact (PVAT+), obtained from normotensive Wistar-Kyoto (WKY) rats and spontaneously hypertensive rats (SHR) at 5th and 12th week of life.

		NA		TES
		pEC50	AUC	AUC
WKY-5w	PVAT−	7.93 ± 0.27	31.31 ± 0.37	8.10 ± 1.36
	PVAT+	6.09±0.16^∗∗∗^	12.08±2.55^∗∗∗^	4.42 ± 0.41^∗^

WKY-12w	PVAT−	6.76 ± 0.21	24.32 ± 2.29	7.60 ± 0.65
	PVAT+	5.86±0.11^∗∗^	13.46±1.82^∗∗^	6.00 ± 0.50

SHR-5w	PVAT−	6.98 ± 0.23	16.49 ± 1.26	3.58 ± 0.43
	PVAT+	5.81±0.21^∗∗^	11.59 ± 1.52^∗^	3.34 ± 0.54

SHR-12w	PVAT−	6.74 ± 0.14	21.54 ± 1.44	8.19 ± 0.60
	PVAT+	6.22±0.99^∗∗^	17.05 ± 1.41	8.05 ± 0.47

NA: noradrenaline; TES: transmural electrical stimulation; pEC50: negative logarithm of half maximal effective noradrenaline concentration; AUC: area under curve. Values represent mean ± SEM of 10 rats. ^∗^*p* < 0.05 and ^∗∗∗^*p* < 0.001 PVAT+ vs. PVAT− in the respective experimental group of rats.

## Data Availability

The data used to support the findings of this study are available from the corresponding author upon request.
